# Radiomics-Based Predictive Nomogram for Assessing the Risk of Intracranial Aneurysms

**DOI:** 10.21203/rs.3.rs-4350156/v1

**Published:** 2024-05-10

**Authors:** Sricharan S. Veeturi, Arshaq Saleem, Diego Ojeda, Elena Sagues, Sebastian Sanchez, Andres Gudino, Elad I. Levy, David Hasan, Adnan H. Siddiqui, Vincent M. Tutino, Edgar A. Samaniego

**Affiliations:** University at Buffalo; University of Iowa; University of Iowa; University of Iowa; Yale University; University of Iowa; University at Buffalo; Duke University; University at Buffalo; University at Buffalo; University of Iowa

**Keywords:** Aneurysms, radiomics, aneurysm wall enhancement

## Abstract

**Background:**

Aneurysm wall enhancement (AWE) has the potential to be used as an imaging biomarker for the risk stratification of intracranial aneurysms (IAs). Radiomics provides a refined approach to quantify and further characterize AWE’s textural features. This study examines the performance of AWE quantification combined with clinical information in detecting symptomatic IAs.

**Methods:**

Ninety patients harboring 104 IAs (29 symptomatic and 75 asymptomatic) underwent high-resolution magnetic resonance imaging (HR-MRI). The assessment of AWE was performed using two different methods: 3D-AWE mapping and composite radiomics-based score (RadScore). The dataset was split into training and testing subsets. The testing set was used to build two different nomograms using each modality of AWE assessment combined with patients’ demographic information and aneurysm morphological data. Finally, each nomogram was evaluated on an independent testing set.

**Results:**

A total of 22 radiomic features were significantly different between symptomatic and asymptomatic IAs. The 3D-AWE Mapping nomogram achieved an area under the curve (AUC) of 0.77 (63% accuracy, 78% sensitivity and 58% specificity). The RadScore nomogram exhibited a better performance, achieving an AUC of 0.83 (77% accuracy, 89% sensitivity and 73% specificity).

**Conclusions:**

Combining AWE quantification through radiomic analysis with patient demographic data in a clinical nomogram achieved high accuracy in detecting symptomatic IAs.

## Introduction

Accurate stratification of rupture risk for intracranial aneurysms (IAs) remains a challenging task. Morphological parameters, such as aneurysm size cutoff of 7 mm, have been suggested for the triage of aneurysms as having high versus low risk of rupture.[1] However, large cohort studies have shown that most ruptured IAs are actually smaller than 7mm in size.[2, 3] Similarly, well-known clinical assessment tools such as the PHASES score[4, 5] were developed to assess risk of rupture. However, further validation efforts have shown a weak correlation between this clinical score and ruptured aneurysm presentation, highlighting the need for more reliable and accurate assessment tools.[6]

Aneurysm wall enhancement (AWE) is increasingly recognized as a promising biomarker of aneurysm instability.[7–9] Histopathological studies have correlated AWE with the presence of inflammatory cells, neovascularization, and vasa vasorum in the aneurysm wall. These histological changes signal degradation of the aneurysmal wall and an increased risk of growth and rupture.[10] To objectively quantify AWE, we have developed a semi-automated pipeline capable of measuring AWE and generating 3D color maps that represent the distribution of signal intensity (SI) within the aneurysm wall.[11]

In this context, radiomics has been also utilized for a voxel-by-voxel analysis of the aneurysm wall. Radiomics is an advanced imaging analysis technique that enables the quantification of SI and textural patterns within a region of interest (ROI). In our previous work we engineered a machine-learning model that leverages radiomic features (RFs) to differentiate between high-risk and low-risk aneurysms based on PHASES scores, achieving an accuracy of 88 to 90%.[12] However, as mentioned earlier, the PHASES score does not have a high accuracy in determining risk of rupture.[13] Therefore, in this study we have used symptomatic status presentation as the primary outcome for developing a predictive model, rather than relying on the PHASES score. This study evaluates the effectiveness of combining AWE measurements obtained through 3D mapping or radiomics, alongside patient demographic information and aneurysm morphological data, in identifying symptomatic IAs.

## Methods

### Patient Population and Aneurysm Characteristics

This study was approved by the institutional review board at the University of Iowa. A prospectively acquired database of patients with IAs imaged between May 2019 to June 2023 with a 3T high resolution magnetic resonance imaging (HR-MRI) was reviewed. Saccular IAs larger than 2 mm were included in the analysis. Aneurysms that exhibited acquisition artifacts, or located in the cavernous segment of the internal carotid artery (ICA), which have a low risk of rupture, were also excluded.[14] Demographic information was retrieved from the electronic medical record. Aneurysms were categorized into symptomatic or asymptomatic. Symptomatic aneurysms encompassed those that had ruptured, presented with cranial nerve neuropathy due to either compression or inflammation, and those associated with sentinel headaches – defined as a sudden, unusually severe headache occurring within two weeks from presentation-.[15] For patients with multiple IAs, the aneurysm deemed as symptomatic was determined by its location, evidence of growth if serial imaging was available, and rupture status. The initial determination of symptomatic status was performed by three investigators (AS, DO, and SS). Any discrepancies from the first review were resolved in consensus with the senior investigator (EAS).

Aneurysm morphological metrics were determined in 3-dimesional rotational angiograms, digital subtraction angiography, computed tomography angiography or magnetic resonance angiography. Aneurysm size was calculated as the maximum diameter,[16] size ratio (SR) was calculated by dividing the maximum aneurysm sac height by the average parent vessel diameter,[17] and aspect ratio (AR) was calculated by dividing the perpendicular aneurysm sac height by the neck width.[18] Additionally, the aneurysm shape was adjudicated as regular or irregular. An aneurysm was considered irregular if it had daughter sacs, blebs, or multiple lobes.[19]

### Image Acquisition and Post-acquisition Processing

A 3T (MAGNETOM Skyra, Siemens) scanner was used to acquire the following sequences: pre-Gd 3D T1, and post-Gd 3D T1 five minutes after the administration of contrast gadolinium (Gd, Gadavist, Bayer Pharmaceuticals, Berlin, Germany) (Supplementary table 1). Isotropic resampling, denoising and co-registration of the images was done prior to segmentation. 3D Slicer (version 5.3.0) was used to generate 3D segmentations of the aneurysm sac, the parent artery and aneurysm wall (shell).[20] The aneurysm shell consisted of a segmentation of solely the aneurysm wall. Additionally, a 2 mm spherical ROI of the corpus callosum (CC) was obtained for individual normalization of the SI.

### 3D AWE Mapping

The segmented aneurysms were analyzed using a proprietary MATLAB pipeline (MathWorks, Natick, Massachusetts, USA) as previously outlined.[21] This post-processing technique extends orthogonal spokes from the aneurysm sac into the aneurysm wall, enabling sampling of the SI from the aneurysm wall. For normalization purposes, the average SI for each aneurysm is then divided by the SI of the CC. This process yields three distinct AWE metrics: 1) 3D Circumferential AWE (3D-CAWE), which calculates the mean SI of the aneurysm wall on T1-post Gd images; 2) Aneurysm-Specific Contrast Uptake AWE (SAWE), defined by the difference in mean SI between T1 post-Gd and T1 pre-Gd images; and 3) Focal AWE (FAWE), which is defined as the proportion of SI in the post-Gd images that is two standard deviations above the mean SI of T1 post-Gd images.[22]

### Radiomic Features Extraction

**Figure 1:**
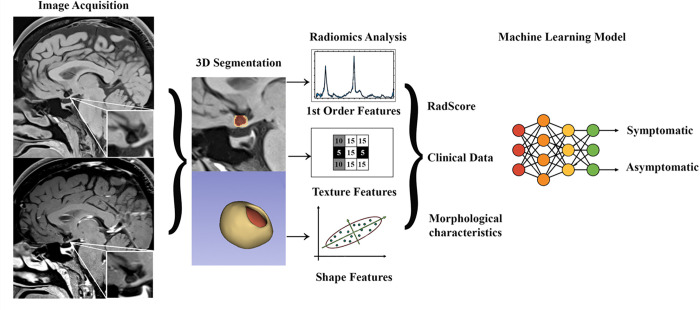
Analysis Pipeline. Images were acquired with a 3T high resolution magnetic resonance imaging with and without contrast gadolinium. Segmentation of the aneurysm wall (shell) and aneurysm sac were performed in 3D Slicer. Radiomic analysis included extraction of enhancement (1^st^ order features), texture and shape features of the aneurysm wall. Transform-based RFs were not included in the figure, as these were not used for analysis. Significant RFs, clinical data and morphological aneurysm characteristics were used to build a machine learning model that accurately identified symptomatic status.

Automatic extraction of RFs from the aneurysm shells in T1-pre and T1-post-Gd was performed using the Python Interactor Module within 3D Slicer.[12] The following RFs were extracted: first-order features related to SI, shape-based features, and textural features.[23] A total of 293 RFs per shell were extracted. Additionally, first order and textural RFs computed from the difference between T1-post and T1-pre-Gd images were obtained (Fig. 1).

### Development of RadScore Model

**Figure 2:**
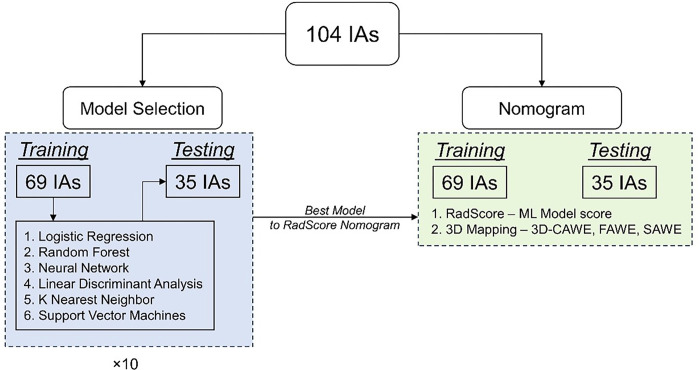
Overall workflow. We split the dataset into training and testing datasets. The training dataset was used to evaluate six different machine learning models. We then evaluate the best machine learning model that can identify symptomatic IAs. We then use the probability of this model as an input to build two nomograms: RadScore-based nomogram and 3D Mapping-based nomogram.

IAs were divided for machine learning analysis into 2/3rd and 1/3rd training and testing subsets (69 and 35 cases respectively). RFs that were significantly different between symptomatic and asymptomatic IAs were selected through univariate analysis of the training set. Collinear RFs were excluded, and the training dataset was used to evaluate six different machine learning models: Logistic Regression, K Nearest Neighbor, Linear Discriminant Analysis, Neural Network, Random Forest and Support Vector Machines (SVM), and a grid search algorithm was utilized for hyper-parameter tuning. The best performing model was determined using a Monte-Carlo cross validation looped over 10 randomizations, and then was used to train a final model to generate a “RadScore” for each aneurysm case (Fig. 2). RadScore represents the probability output of the final model; it’s a composite representation of the values of the RFs obtained from the T1-pre and T1-post-Gd sequences. The RadScore for the testing cases was also determined using the final model on the 35 hold out IAs. Ultimately, we generated a training set and an independent testing dataset with a composite RadScore to build a subsequent nomogram.

### Development and Evaluation of Nomograms

Patient demographics and IA morphological metrics, such as: size, SR and AR were used to generate clinical nomograms based on the 69 IAs from the training data set. These clinical and morphological data were combined with 3D AWE mapping metrics and the RadScore results to construct two nomograms for prediction of IA symptomatic status. An open-source python library (pyNomo) was used to build nomograms based on an underlying logistic regression model.[24] The performance of the nomograms was evaluated on the independent 35 testing cases.

### Statistical Analysis

The demographic data was analyzed using IBM SPSS Statistics for Windows, version 27.0. Shapiro-Wilk tests were performed on continuous data to assess for normality. Normally distributed variables are listed as mean ± standard deviation (SD) and non-normally distributed variables are represented as median (interquartile range). Categorical data are listed as frequency (percentage). Comparisons between normally distributed data were performed by using the Student’s t-tests and Pearson correlations. Comparisons involving non-normally distributed data were conducted using Mann-Whitney U test and Spearman’s correlation. Comparisons between unpaired categorical data were performed using Pearson’s X^2^ test or Fisher’s exact test. The alpha value was set at 0.05.

For radiomics univariate analysis for building the RadScore model, a python package (scipy v 1.11.3) was used. [25] Each RF was assessed for normality using a Shapiro-Wilk test. For normally distributed parameters, differences between symptomatic and asymptomatic aneurysms were then computed using the student t-test. For non-normally distributed variables, a Mann-Whitney U test was used.

## Results

### Demographics and Aneurysm Morphology

A total of 255 patients who underwent the HR-MRI protocol were studied. One hundred and sixty-five patients were excluded as they had aneurysms that were either fusiform (n = 44), thrombosed (n = 11), smaller than 2 mm (n = 20), or close to the cavernous sinus (n = 2). Additionally, aneurysms with poor image quality (n = 88) were excluded. A total of 90 patients with 104 aneurysms (29 symptomatic and 75 asymptomatic) were included (Supplementary table 2). Sentinel headache was the most frequent symptom in 83% of the symptomatic patients (n = 24). Aneurysm rupture was confirmed in 31% of the symptomatic patients (n = 9) and 24% had cranial nerve palsy (n = 7) (Supplementary table 3). The average age was 67 years old in the asymptomatic group and 56 years old in the symptomatic group, with women comprising 81.3% (n = 61) of the asymptomatic group and 86.2% (n = 25) of the symptomatic group. Asymptomatic aneurysms were in the middle cerebral artery (MCA, n = 20, 26%), ICA (n = 19, 24%) and anterior communicating artery (ACOM) complex (n = 16, 23%); whereas symptomatic aneurysms were in the ACOM complex (n = 8, 28%), MCA (n = 7, 24% and ICA (n = 6, 21%). Twenty-two patients (24%) had more than one aneurysm. Asymptomatic aneurysms had a smaller size, lower SR, lower AR and were less irregular than symptomatic aneurysms (median size of 4.9 mm, IQR 3.20 vs 6.90 mm, IQR = 4.45, p = 0.017; SR of 1.91, IQR = 1.66 vs 3.12, IQR = 1.64, p = 0.011; AR of 1.18, IQR = 0.94 vs 1.53, IQR = 1.04, p = 0.007; and 49% vs 59% irregular IAs).

### Radiomic Features are Significantly Associated with Symptomatic Presentation

A total of 87 RFs were significantly different between asymptomatic and symptomatic IAs (supplementary table 4). Shape-based RFs such as volume, maximum diameter and least axis length were significantly higher in symptomatic IAs. First-order RFs were significantly higher in symptomatic IAs in pre-Gd, post-Gd T1 scans, as well as the difference between post and pre-Gd T1 scans. Texture-based RFs derived from pre-Gd T1 sequences, such as large area high gray level emphasis (LAHGLE) was significantly higher in symptomatic IAs. Textural RFs derived from post-Gd T1 sequences exhibited higher large dependence high gray level emphasis (LDHGLE) as well as long run high gray level emphasis (LRHGLE) in symptomatic IAs. Finally, total textural RFs such as energy and variance were significantly higher in symptomatic IAs after computing the difference between the post- and pre-Gd T1 sequences.

### Composite Radiomics-based Score for Risk Evaluation

After excluding collinear variables, a total of 22 RFs significantly different between symptomatic and asymptomatic cases were used to train the machine learning models. The linear discriminant analysis, logistic regression, K-nearest neighbor and the SVM models had comparable accuracies, however, the logistic regression model had the highest sensitivity and the highest AUC amongst all models (supplemental Fig. 1 and supplementary table 5).

### Clinical Nomograms for Symptomatic IA Status Prediction.

**Figure 3:**
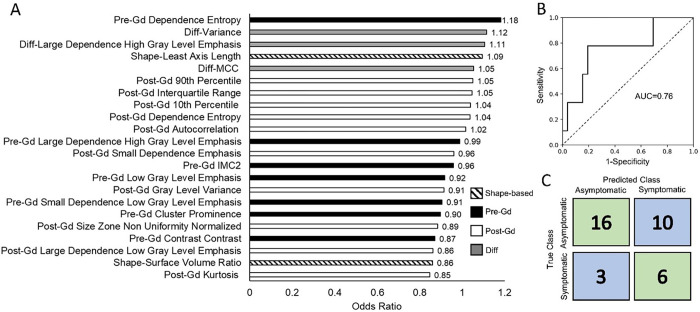
Final trained RadScore model. The bar plot shows odds ratios for all the radiomics features used in the RadScore model in descending order of importance. The overall AUC was 0.76 and the accuracy was 63% (67% sensitivity and 62% specificity).

The 3D Mapping based nomogram achieved an accuracy of 63%, sensitivity of 78%; specificity of 58% and AUC of 0.77 in predicting symptomatic IAs. The final trained RadScore model, before adding the clinical information, had an AUC was 0.76 (63% accuracy, 67% sensitivity and 62% specificity) (Fig. 3).

**Figure 4:**
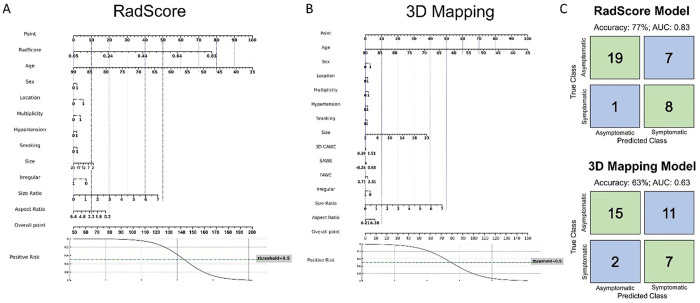
Nomograms for RadScore (Composite Radiomics-based score) and 3D mapping models. Two nomograms were constructed using RadScore, AWE metrics from 3D mapping pipeline and patient demographics (gender = female 1 and male 0; location = 0 for low-risk location MCA or ICA, and = 1 for high-risk location ACom or posterior circulation; multiplicity = 0 for a single aneurysm in the patient and 1 for more than one aneurysm; hypertension = 0 for no hypertension and 1 for hypertension; and smoking = 0 for never smoked and 1 for past or current smoker) and IA characteristics (size, SR, AR, irregularity = 0 for smooth regular shape and 1for multi-lobulated irregular shape or has blebs). We observed that the RadScore-based nomogram had a higher accuracy, sensitivity and specificity compared to the 3D mapping pipeline-based nomogram.

The RadScore-based nomogram after including demographic variables achieved a higher accuracy, sensitivity, and specificity than the 3D mapping-based nomogram with an AUC of 0.83 (77.1% accuracy, 89% sensitivity, and 73% specificity, Fig. 4).

## Discussion

A total of 87 RFs were significantly different between asymptomatic and symptomatic IAs and after eliminating collinear variables, 22 RFs were used to compute a composite “RadScore”. The RadScore nomogram had a better performance than the 3D mapping-based nomogram in identifying symptomatic IAs. This nomogram presents a comprehensive model combining clinical data, IA morphology metrics and AWE assessment through radiomics. These results underscore the need for a comprehensive assessment in the triage of unruptured IAs, with inclusion of known clinical risk factors, aneurysm characteristics and advanced post-imaging processing tools.

Twelve shape-based RFs were significantly different between asymptomatic and symptomatic IAs. RFs such as major axis length, maximum 3D diameter, surface area and mesh volume were significantly higher in symptomatic IAs. These RFs translate to different size metrics of the aneurysm, with larger values suggesting the presence of larger size IAs, confirming that in general larger aneurysms are more likely to be symptomatic.

First order RFs from pre-Gd sequences, energy and total energy were significantly higher in symptomatic IAs. Energy represents the magnitude of voxel intensities, and a higher value suggests that voxels in the wall of symptomatic IAs have higher intensity values. Similarly, the non-uniformity computed through the gray level size zone matrix (GLSZM) and gray level run length matrix (GLRLM), which represent the connectivity of similar intensity voxels, were also higher in symptomatic IAs. Since increased intensity is sometimes suggestive of thickened tissue, elevated values of RFs that evaluate SI suggest that symptomatic IAs tend to have thicker walls.[26] This thickening can be interpreted as areas of inflammation, whereas thinner sections might be suggestive of the presence of blebs. Research by Liu et al. has shown that IAs with thicker walls are often larger and exhibit enhancement, both of which are markers of aneurysm instability.[27]

Radiomics analysis of post-Gd T1 sequences showed that first order RFs like mean, median, energy, and 90th percentile, among others, were higher in symptomatic IAs, indicating a higher degree of contrast uptake by the aneurysm wall. A high value of SI in post-Gd sequences suggests heterogeneous biological processes within the aneurysm wall, such as microbleeds or thrombosis.[28, 29] Similarly, we also observed higher value of GLRLM, long run high gray level emphasis (LRHGLE), GLSZM and large area high gray level emphasis (LAHGLE). These metrics quantify the connectivity of high intensity voxels and proportion of high intensity area in the IA wall. A high value of GLRLM LRHGLE suggests long runs of high-intensity voxels which could be suggestive of the presence of vasa vasorum. Similarly, a high value of GLSZM LAHGLE indicates large areas with high intensity, and high contrast absorption. These textural features of enhancement suggest a heterogenous environment within the aneurysm wall, with different potential biological processes affecting the wall and therefore the SI captured at a voxel level with RFs.

Quantitative metrics obtained through vessel wall imaging data can be used for risk stratification of IAs. [30–32] Recently, a study by Fu et al. demonstrated that in a cohort of 341 IAs (248 asymptomatic and 93 symptomatic) wall enhancement index (a metric quantifying the difference between normalized signal intensity of post and pre-Gd MRIs) as well as the pattern of wall enhancement could accurately predict symptomatic IAs with an AUC of 0.91 and sensitivity of 95.7% and a specificity of 73.4%.[33] Similarly, Raghuram et al. showed that in a cohort of 93 IAs (73 asymptomatic and 20 symptomatic), presence of 3D-CAWE was positively associated with IA symptomatic presentation.[22] This study combines clinical and morphological data with objective assessment of AWE through radiomics and 3D mapping to construct clinical nomograms for symptomatic IA prediction. The RadScore based nomogram was the best performing model, with 77.1% accuracy (89% sensitivity; 735 specificity and 0.83 AUC) indicating that radiomics may be better for evaluating the IA wall compared to 3D AWE mapping.

This study has several limitations. First, the sample size of the external dataset for this analysis was small. Second, we used symptomatic presentation of IAs as our end point for the analysis of high-risk IAs, and although this is more accurate than PHASES, or size-based thresholds, true risk can only be obtained through longitudinal data. Finally, changes in resolution and acquisition metrics of the 3T-MRI may affect the radiomics based model, and studies to standardize these parameters among different scanners are needed.

## Conclusion

In this study, we used a radiomics based pipeline to assess AWE and characterize the heterogeneous aneurysm wall environment. A Radiomics-based composite score was developed to identify symptomatic high-risk IAs and build clinical nomograms. Integrating clinical information, aneurysm morphological data and radiomic information rendered the best predictive model of IA symptomatic status. These findings emphasize the necessity of a comprehensive assessment, including advanced post-processing image analysis, for accurate prediction of symptomatic status in IAs.

## Data Availability

Clinical information and images were obtained from the medical records at the University of Iowa Hospitals and Clinics. The information was de-identified during the analysis.
